# Supplementary material for: Early exposure to farm dust in an allergic airway inflammation rabbit model: Does it affect bronchial and cough hyperresponsiveness?
Source: PLoS One. 2023 Jan 27;18(1):e0279498. doi: 10.1371/journal.pone.0279498 (PMC9882901; doi:10.1371/journal.pone.0279498)
Supplement: S1 Table — (DOCX) [file pone.0279498.s001.docx]

**Supporting Table 1**

**S1 Table. Correlation between quantitative variables** (Spearman’s rank correlation coefficient)

|  |  | Weight | DR maximal intensity | CR maximal intensity | ER maximal intensity | Initial respiratory resistance | PD50 | PD100 |
| --- | --- | --- | --- | --- | --- | --- | --- | --- |
| Age | rho | 0.70516 | 0.20075 | -0.03218 | 0.1974 | -0.05592 | 0.57117 | 0.58756 |
|  | p-value | <0.0001 | 0.2706 | 0.876 | 0.3786 | 0.7651 | 0.001 | 0.001 |
|  | N | 38 | 32 | 26 | 22 | 31 | 30 | 28 |
| Weight | rho |  | 0.17657 | 0.20075 | 0.14585 | -0.16376 | 0.3445 | 0.40246 |
|  | p-value |  | 0.3337 | 0.3254 | 0.5172 | 0.3787 | 0.0623 | 0.0337 |
|  | N |  | 32 | 26 | 22 | 31 | 30 | 28 |
| DR maximal intensity | rho |  |  | 0.85983 | 0.63298 | 0.25567 | 0.12589 | 0.15009 |
|  | p-value |  |  | <0.0001 | 0.0016 | 0.1807 | 0.5233 | 0.4643 |
|  | N |  |  | 26 | 22 | 29 | 28 | 26 |
| CR maximal intensity | rho |  |  |  | 0.22647 | 0.05565 | -0.167 | -0.11124 |
|  | p-value |  |  |  | 0.399 | 0.7962 | 0.4463 | 0.6221 |
|  | N |  |  |  | 16 | 24 | 23 | 22 |
| ER maximal intensity | rho |  |  |  |  | 0.06316 | 0.53767 | 0.57108 |
|  | p-value |  |  |  |  | 0.7973 | 0.0214 | 0.0166 |
|  | N |  |  |  |  | 19 | 18 | 17 |
| Initial respiratory resistance | rho |  |  |  |  |  | -0.09811 | -0.1538 |
|  | p-value |  |  |  |  |  | 0.606 | 0.4346 |
|  | N |  |  |  |  |  | 30 | 28 |
| PD50 | rho |  |  |  |  |  |  | 0.98962 |
|  | p-value |  |  |  |  |  |  | <0.0001 |
|  | N |  |  |  |  |  |  | 27 |
