# Supplementary material for: Early exposure to farm dust in an allergic airway inflammation rabbit model: Does it affect bronchial and cough hyperresponsiveness?
Source: PLoS One. 2023 Jan 27;18(1):e0279498. doi: 10.1371/journal.pone.0279498 (PMC9882901; doi:10.1371/journal.pone.0279498)
Supplement: S2 Table — (DOCX) [file pone.0279498.s002.docx]

**Supporting Table 2**

**S2 Table. Defensive reflexes provoked by nebulization of acid citric aerosol**

|  |  | Control Group |  | Farm Group |
| --- | --- | --- | --- | --- |
| **Acid citric threshold to elicit at least one defensive reflex** |  |  |  |  |
| 0.2 |  | 0 |  | 3 |
| 0.4 |  | 3 |  | 0 |
| 0.8 |  | 0 |  | 1 |
| 1.6 |  | 0 |  | 0 |
| None |  | 16 |  | 14 |
| **Cumulative number of defensive reflexes** |  |  |  |  |
| None |  | 16 |  | 14 |
| 1-2 |  | 3 |  | 2 |
| 3-5 |  | 0 |  | 0 |
| >5 |  | 0 |  | 2 |
