# Supplementary material for: Early exposure to farm dust in an allergic airway inflammation rabbit model: Does it affect bronchial and cough hyperresponsiveness?
Source: PLoS One. 2023 Jan 27;18(1):e0279498. doi: 10.1371/journal.pone.0279498 (PMC9882901; doi:10.1371/journal.pone.0279498)
Supplement: S1 File — (DOCX) [file pone.0279498.s003.docx]

**Supporting methods**

**Sensitization and provocation of airway inflammation with ovalbumin**

All rabbits were sensitized around their 12^th^ week of life by two intraperitoneal (IP) injections of a 1 mL suspension containing 0.1 mg ovalbumin (OVA) (Sigma-Aldrich, Saint Quentin Fallavier, France) and 10 mg aluminum hydroxide (Sigma-Aldrich, Saint Quentin Fallavier, France) in sterile saline (0.9% NaCl) on days 0 and 13 (1). The solutions were suspended for a 30-minute agitation on ice before the IP injections.

From day 23, that is, 10 days after the end of sensitization, the rabbits received an OVA aerosol for 20 minutes each day for four days. The OVA aerosols were administered using an ultrasonic nebulizer (LS 290, SYSTAM^®^) that produced droplets with a mass media aerodynamic diameter of 3.5 µm (60% of particles had a size between 1 and 5 µm) connected to a custom-built device created in our laboratory. The system was a closed Plexiglas chamber (33 x 33 x 51.5 cm) consisting of a head chamber isolated from a body chamber. The rabbits were positioned in order to permit proximity between the rabbit’s nose and the opening for aerosol diffusion. To achieve the aerosol challenges, the rabbits were placed in the restraining system that had an opening for tubing connected to a nebulizer containing 20 mL of an OVA solution (2.5 mg.mL^-1^ OVA in saline). A 20-minute nebulization allowed for the aerosolization of almost 40 mg of OVA. All aerosols were performed in a class I safety enclosure for safety reasons. The rabbits were exposed to the last OVA aerosol 48 hours before the mechanical and chemical cough challenges. During the five days preceding the allergen aerosol administration, five habituation sessions of five minutes each using a saline solution were performed in order to reduce the stress associated with handling and the movement restriction imposed by the nebulization system.

**Chemical stimulation in conscious rabbits**

A saline solution followed by four citric acid (Sigma-Aldrich, Saint Quentin Fallavier, France) solutions with different concentrations (0,2M, 0,4M, 0,8M and 1,6M) were nebulized using an ultrasonic nebulizer (LS 290, SYSTAM®) during one minute for each in the same Plexiglas chamber that the one used for OVA aerosols. Each aerosol was separated by at least one minute. Cough response to nebulization of citric acid was quantified by the number of defensive reflex (DR) counted by an observer at each concentration. Chemical threshold was defined as the lower concentration of acid citric solution that elicit a DR. The cumulative number of DR during the four different solutions was calculated.

**Anesthesia and animal preparation**

**Anesthesia, analgesia, and euthanasia**

Analgesia was induced by an intra-muscular injection of buprenorphine (0.02 mg.kg^-1^). Fifteen minutes after pre-medication, anesthesia was induced with propofol (3 mg.kg^-1^) (Ceva Santé Animale, Libourne, France) and ketamine (3 mg.kg^-1^) (Virbac, France; Carros, France) injected through the ear vein. The anesthesia was then maintained by a continuous perfusion of propofol (0.8 mg.kg^-1^.min^-1^) and ketamine (0.2 mg.kg^-1^.min^-1^). Monitoring of analgesia and anesthetic depth was performed in 15-minute intervals by assessing the change in physiological parameters (heart and respiratory rate) and the absence of withdrawal responses to compressive ear and toe pinches. The perfusion rate was adapted according to this monitoring.

At the end of the experiment, the animals were sacrificed by intravenous injection of 3 mL of Euthasol (400 mg of pentobarbital per milliliter, Dechra).

**Animal preparation**

The anesthetized animal was placed in the supine position. Its rectal temperature was continuously monitored with an electrical thermistor (Physitemp Instruments, YSI 402 Clifton, NJ, USA) and maintained at 38℃ using a warm water circulation pad. The electromyographic activity of the rectus abdominis muscle was measured by the insertion of bipolar insulated fine stainless steel wire electrodes (A-M Systems INC, Sequim, WA 98,382) introduced under visual control into either the transversus abdominis or external oblique abdominal muscles according to Basmajian and Stecko (2) to further differentiate the active expiration from the augmented breath (3). An upper cervical tracheotomy allowed the insertion of a tracheal cannula that was connected to the mechanical stimulation apparatus and a pneumotachograph (No. 0 Fleisch pneumotachograph with a linear range of ± 250 mL.s^-1^). The pneumotachograph was calibrated before each experiment using a 20 ml calibration syringe.
