# Supplementary material for: Early exposure to farm dust in an allergic airway inflammation rabbit model: Does it affect bronchial and cough hyperresponsiveness?
Source: PLoS One. 2023 Jan 27;18(1):e0279498. doi: 10.1371/journal.pone.0279498 (PMC9882901; doi:10.1371/journal.pone.0279498)
Supplement: S2 File — (DOCX) [file pone.0279498.s004.docx]

**Supporting results**

Among the 37 rabbits challenged by acid citric nebulization, only 8 rabbits (21.6%) had at least one DR to chemical stimulation without statistical difference between groups (3 in C and 4 in F, p=0.693). Chemical threshold and cumulative number of DR provoked by nebulization of acid citric are described in Table S2. Chemical threshold was different between groups (p=0.036) whereas the cumulative number of DR was not (p=0.567).
